# Deficiency in Tissue Non-Specific Alkaline Phosphatase Leads to Steatohepatitis in Mice Fed a High Fat Diet Similar to That Produced by a Methionine and Choline Deficient Diet

**DOI:** 10.3390/ijms22010051

**Published:** 2020-12-23

**Authors:** Reyes Gámez-Belmonte, Mireia Tena-Garitaonaindia, Cristina Hernández-Chirlaque, Samir Córdova, Diego Ceacero-Heras, Fermín Sánchez de Medina, Olga Martínez-Augustin

**Affiliations:** 1 Department of Pharmacology, CIBERehd, School of Pharmacy, Instituto de Investigación Biosanitaria ibs.GRANADA, University of Granada, 18071 Granada, Spain; MariadelosReyes.GamezBelmonte@uk-erlangen.de; 2 Department of Biochemistry and Molecular Biology 2, CIBERehd, School of Pharmacy, Instituto de Investigación Biosanitaria ibs.GRANADA, Instituto de Nutrición y Tecnología de los Alimentos José Mataix, University of Granada, 18071 Granada, Spain; mireiat94@gmail.com (M.T.-G.); cristinahech@hotmail.com (C.H.-C.); clamirja25@correo.ugr.es (S.C.); diegocastulo98@gmail.com (D.C.-H.); omartine@ugr.es (O.M.-A.)

**Keywords:** methionine and choline diet, metabolic syndrome, biliary acids, tissue non-specific alkaline phosphatase, steatohepatitis

## Abstract

The liver expresses tissue-nonspecific alkaline phosphatase (TNAP), which may participate in the defense against bacterial components, in cell regulation as part of the purinome or in bile secretion, among other roles. We aimed to study the role of TNAP in the development of hepatosteatosis. TNAP^+/−^ haplodeficient and wild type (WT) mice were fed a control diet (containing 10% fat *w/w*) or the same diet deficient in methionine and choline (MCD diet). The MCD diet induced substantial weight loss together with hepatic steatosis and increased alanine aminotransferase (ALT) plasma levels, but no differences in IL-6, TNF, insulin or resistin. There were no substantial differences between TNAP^+/−^ and WT mice fed the MCD diet. In turn, TNAP^+/−^ mice receiving the control diet presented hepatic steatosis with alterations in metabolic parameters very similar to those induced by the MCD diet. Nevertheless, no weight loss, increased ALT plasma levels or hypoglycemia were observed. These mice also presented increased levels of liver TNF and systemic resistin and glucagon compared to WT mice. The phenotype of TNAP^+/−^ mice fed a standard diet was normal. In conclusion, TNAP haplodeficiency induces steatosis comparable to that produced by a MCD diet when fed a control diet.

## 1. Introduction

Non-alcoholic fatty liver disease (NAFLD), defined by the accumulation of fat in the liver in the absence of significant alcohol intake, encompasses a spectrum of liver diseases that can be further characterized as either non-alcoholic fatty liver (NAFL) or non-alcoholic steatohepatitis (NASH), based on the presence or absence of inflammation and liver cell injury. Because of its high incidence/prevalence, and its possible progression to cirrhosis and ultimately to hepatic cancer, NAFLD is increasingly attracting attention [1]. In addition, there is a close link between metabolic syndrome and NAFLD [2].

Alkaline phosphatases are a group of isoenzymes that in humans include the intestinal (IAP), tissue non-specific (TNAP), fetal and placental isoforms [3]. These enzymes share substrates and a common mechanism of action. Among them, TNAP comprises three isoforms (liver, bone and kidney), identical in their final sequence but differing in their glycosylations. TNAP is a pleiotropic enzyme expressed in multiple cell types and tissues, including liver, bone, brain, intestine, lymphocytes, macrophages or neutrophils. Multiple substrates, such as bacterial components (LPS), nucleotides, phosphorylcholine (PC), phosphoryl-ethanolamine or pyridoxal-5′-phosphate, have been described for TNAP, and as a consequence, it has a role in multiple systemic processes, including bone mineralization, vitamin B_6_ metabolism and neurogenesis [4]. Mutations in the encoding gene ALPL are the cause of hypophosphatasia.

In recent years, TNAP has been shown to exert different anti-inflammatory effects depending on its actions on the innate and adaptive immune system. Two main mechanisms of action have been described. An increase in the production of IL-6 and TNF has been observed in neutrophils from heterozygous TNAP^+/−^ mice stimulated with LPS, indicating an anti-inflammatory effect of the enzyme, possibly dependent on LPS dephosphorylation [5]. Another mechanism for TNAP to influence inflammation is the regulation of purinergic signaling. In this regard, a recent study shows that TNAP inhibition in neutrophils significantly exacerbates ATP-associated activation and secretion of IL-1β [6]. We have shown that TNAP is required for T cell activation and TNAP^+/−^ T cells exhibit a decreased colitogenic potential in vivo [7].

NAFLD and other features of metabolic syndrome, including obesity or diabetes, are linked to subclinical inflammation, induced by alterations in the intestinal barrier, resulting in increased levels of circulating bacterial antigens. Several studies have shown the association of IAP with metabolic syndrome. In this regard, the administration of IAP to mice fed a high-fat diet prevents endotoxemia and the associated metabolic syndrome [8], while *Akp3* (duodenal AP) knock-out mice show faster weight gain [9], visceral fat accumulation and hepatic steatosis, as well as insulin resistance. Similarly, IAP deficiency has been related to a higher lipid absorption in animals fed a high-fat diet [8]. The effects of IAP on metabolic syndrome have been attributed to its ability to dephosphorylate LPS and possibly other bacterial molecules. Since TNAP shares anti-inflammatory actions and mechanisms with IAP and is involved in purinergic signaling, we hypothesized that it could have a role in preventing hepatosteatosis. TNAP KO mice are only viable for a few days; they die before weaning and show vitamin B_6_-sensitive epilepsy and impaired bone mineralization. TNAP^+/−^ haplodeficient mice are, however, viable, although they exhibit an altered immune phenotype as noted.

TNAP is also known to dephosphorylate PC, to obtain choline to be absorbed in the intestine, and is needed for the synthesis of phospholipids, acetylcholine and trimethylglycine. Choline deficiency inhibits the synthesis of phosphatidylcholine required for very low-density lipoprotein (VLDL) production and induces lipid accumulation in the liver [10,11]. Here we explore the effect of the absence of an allele of TNAP (i.e., TNAP haplodeficiency) on the development of fatty liver by methionine- and choline-deficient diet (MCD) with a 10% *w/w* content of fat (22.1% Kcal from fat). In turn, the deficiency of methionine decreases the biosynthesis of glutathione, leading to oxidative stress, which in turn contributes to liver damage [12]. We found that TNAP^+/−^ responded to the MCD diet just like WT mice but developed fatty liver with the control diet, underscoring a role of TNAP in the susceptibility to this dietary challenge.

## 2. Results

### 2.1. The Absence of a Single Alpl Allele Does Not Modify the Response of Mice to the MCD Diet

**Figure 1:**
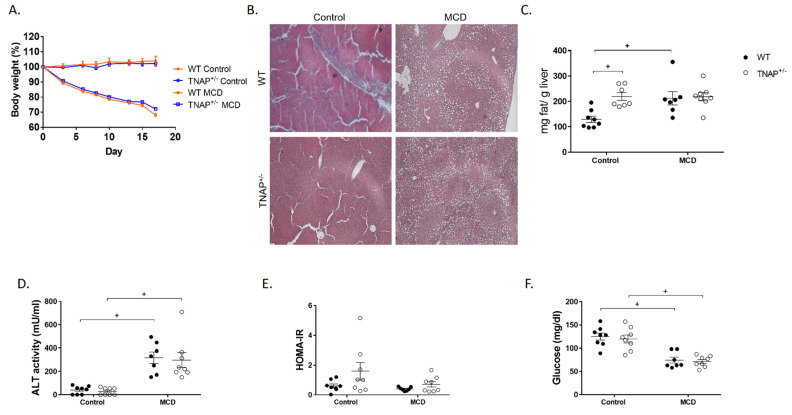
Main parameters of the methionine- and choline-deficient diet (MCD) model. Body weight evolution (**A**), liver histology (**B**), fat liver content (**C**), plasma ALT activity (**D**), glucose concentration in plasma (**E**) and HOMA-IR index (**F**) of wild type (WT) and TNAP heterozygous mice (TNAP^+/−^) fed a control diet (10% *w/w* fat, control) or the same diet deficient in choline and methionine (MCD) ^+^ *p* < 0.05.

**Figure 2:**
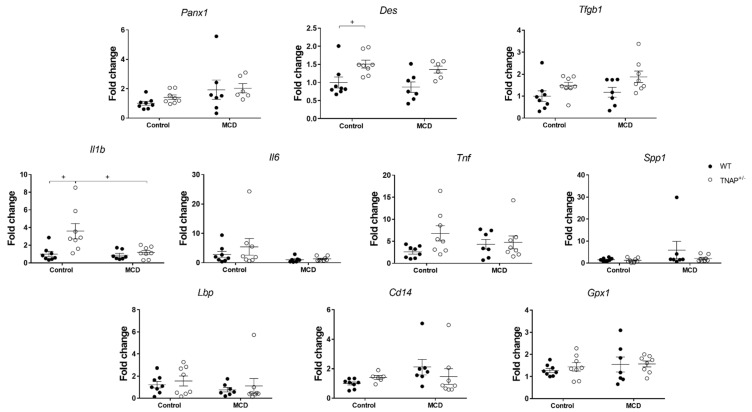
Hepatic expression of fibrosis and inflammatory genes in wild-type (WT) and TNAP heterozygous mice (TNAP^+/−^) a control diet (10% *w/w* fat, control) or the same diet deficient in choline and methionine (MCD). Fold change of qRT-PCR data are shown. ^+^ *p* < 0.05.

**Figure 3:**
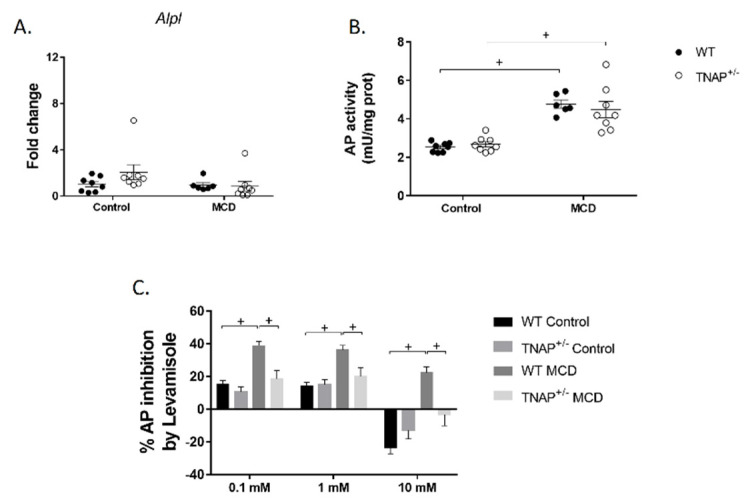
Hepatic alkaline phosphatase expression (**A**), activity (**B**) and sensitivity to levamisole (**C**) of wild-type (WT) and TNAP heterozygous mice (TNAP^+/−^) fed a control diet (10% *w/w* fat, control) or the same diet deficient in choline and methionine (MCD). For AP expression, fold change qRT-PCR data are shown. ^+^ *p* < 0.05.

As expected, feeding the MCD diet resulted in sustained body weight loss in wild-type mice for up to 17 days, when the experiment was halted for ethical reasons, as MCD-fed mice had lost more than 20% of their body weight (Figure 1A). At this time, the expected development of fatty liver was confirmed, as shown by steatosis (Figure 1B,C) and increased alanine aminotransferase (ALT) circulating levels (Figure 1D). Nevertheless, no alteration in the expression of pannexin 1 (*Panx1*, recently related to inflammation in non-alcoholic steatohepatitis and to liver damage [13]) or the fibrosis markers transforming growth factor β1 (*Tgfb1*) or desmin (*Des*) was observed (Figure 2). Accordingly, no hepatic inflammation was shown, since liver mRNA levels of IL-1β (*Il1b*), IL-6 (*Il6*), TNF (*Tnf*) and osteopontin (*Spp1*) were comparable to those of WT mice fed the control diet (Figure 2). The hepatic expression of other inflammation-related markers, LPS binding protein (*Lbp*), *Cd14* or glutathione peroxidase 1 (*GPx1*), was also unchanged (Figure 2). Interestingly, alkaline phosphatase activity and its sensitivity to levamisole were increased in WT mice fed the MCD diet, consistent with a change in isoform (Figure 3). In general, these results indicate the induction of steatosis but no inflammation in the liver of WT animals fed an MCD diet.

**Figure 4:**
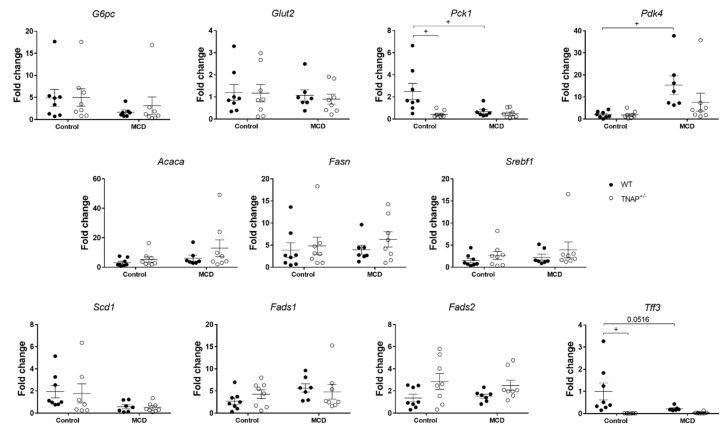
Hepatic expression of glucidic and lipidic metabolism genes in wild-type (WT) and TNAP heterozygous mice (TNAP^+/−^) fed a control diet (10% *w/w* fat, control) or the same diet deficient in choline and methionine (MCD). Fold change of qRT-PCR data are shown. ^+^ *p* < 0.05.

Glucidic metabolism genes were also assessed. No changes in the expression of glucose-6-phosphatase catalytic subunit (*G6pc*) or glucose transporter 2 (*Glut2*) were observed in WT mice fed the MCD diet, while a deep inhibition of liver phosphoenolpyruvate carboxykinase 1 (*Pck1*), the key gluconeogenic enzyme, as well as induction of pyruvate dehydrogenase kinase 4 *(Pdk4)*, were noted (Figure 4). Pyruvate dehydrogenase kinase 4 inhibits the pyruvate dehydrogenase complex and therefore the synthesis of acetyl-CoA from pyruvate.

When liver fatty acid metabolism was studied, the expression of key enzymes in fatty acid synthesis, namely acetyl-CoA carboxylase (*Acaca*) and fatty acid synthase (*Fasn)*, of the transcription factor steroid regulatory element-binding protein 1 (*Srebf1)*, and of fatty acid desaturase genes (*Scd1*, *Fads1* or *Fads2*), was unchanged (Figure 4). Nevertheless, an almost significant decrease in *Tff3* (*p* = 0.056), recently described as a regulator of lipid metabolism [14], was observed (Figure 4).

**Figure 5:**
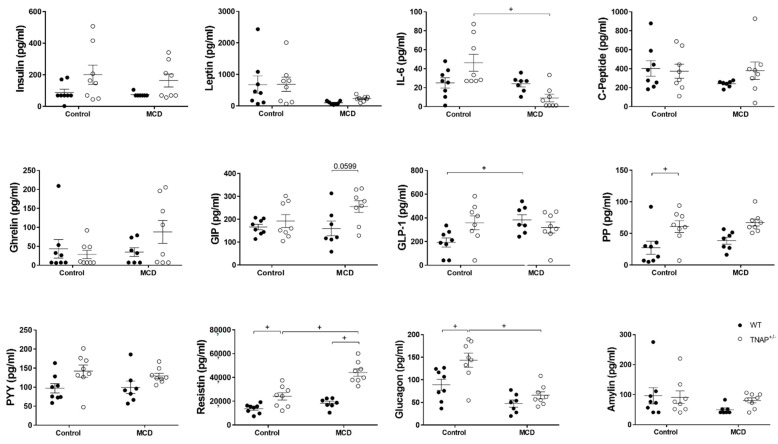
Plasma concentration of inflammation-, obesity- and steatosis-related markers in wild-type (WT) and TNAP heterozygous mice (TNAP^+/−^) fed a control diet (10% *w/w* fat, control) or the same diet deficient in choline and methionine (MCD). Results from the Metabolic Hormone Expanded Panel multiplex are shown. ^+^ *p* < 0.05.

The MCD diet induced hypoglycemia with no change in insulin levels; therefore, the HOMA-IR index was not affected (Figure 1E and Figure 5). Plasma levels of leptin, IL-6, C-peptide, ghrelin, gastric inhibitory peptide (GIP), pancreatic peptide (PP), peptide YY (PYY), resistin and amylin were also unchanged (Figure 5). Only the levels of glucagon-like peptide 1 (GLP-1) were shown to be increased, while those of glucagon were reduced, just short of significance (*p* = 0.08).

The assessment of intestinal parameters showed that weight-to-length ratio was decreased in the colon, ileum and jejunum. Neither alkaline phosphatase activity nor the expression of Alpl (encoding TNAP) were altered by the administration of the MCD diet (Figure S1A/B). Only, as described in liver, sensitivity to levamisole was augmented. Intestinal inflammatory or tight junction genes such as *S100a8*, *Cldn4* and *Tjp1* remained unchanged (Figure S1C/D).

The effects of the MCD diet on TNAP^+/−^ mice were very similar to those on wild type mice, and the phenotypes were almost indistinguishable. The exception to this rule was the increased levels of resistin and an almost significant increase in GIP (*p* = 0.06) in the plasma of TNAP^+/−^ mice (Figure 5), plus the lower sensitivity to levamisole of alkaline phosphatase both in the intestine and liver, which was very similar to that of control-diet-fed groups (Figure 3C).

### 2.2. Alpl Haplodeficiency Induces Hepatic Fat Accumulation in Mice Fed a Control Diet

Somewhat unexpectedly, when fed the control diet, the phenotype of TNAP^+/−^ mice differed considerably from that of the WT group. Thus systemic levels of glucagon, resistin, GLP-1 and PP were increased in the TNAP^+/−^ group (Figure 5). Insulin and PYY showed a similar pattern but without reaching significance. An almost significant systemic increase in IL-6 (*p* = 0.076) was also observed. Remarkably, liver fat was augmented in TNAP haplodeficient mice and was comparable to that of MCD-diet-fed animals of both genotypes (Figure 1). However, steatosis presented with microdeposits at the histological level, while larger fat deposits were observed in mice given the MCD diet with either genotype (Figure 1B). In turn, there was no change in body weight, fasting blood glucose, insulin or HOMA index, although values tended to be higher in TNAP^+/−^ mice in the latter case (Figure 1 and Figure 5).

This phenotype was not anticipated, since TNAP^+/−^ mice show no obvious phenotype at the hepatic level, or indeed globally, in basal conditions. Since the control diet used includes a higher-than-normal fat content, we decided to analyze the hepatic status under standard feeding conditions, i.e., using a regular chow diet. In these conditions, no steatosis was observed (Figure S2A), indicating that the interaction between genotype and the control diet is responsible for the observed phenotype.

### 2.3. Characterization of Steatosis in Alpl Haplodeficient Mice

In addition to steatosis, RT-qPCR analysis revealed an increased expression of hepatic *Il1b* and an almost significant increase in *Tnf* (*p* = 0.08). No other inflammation-related genes were affected (*Il6*, *Lbp*, *Cd14*, *Spp1* or *Gpx1*) (Figure 2). In turn, desmin gene expression was increased in the liver of haplodeficient mice, consistent with possible fibrosis, and *Tgfb* expression exhibited a very similar pattern, without reaching significance. Plasma ALT activity and *Panx1* mRNA level were similar to those of WT mice fed the same diet (Figure 1D and Figure 2). Taken together, these results are consistent with mild inflammation with no substantial hepatic injury.

**Figure 6:**
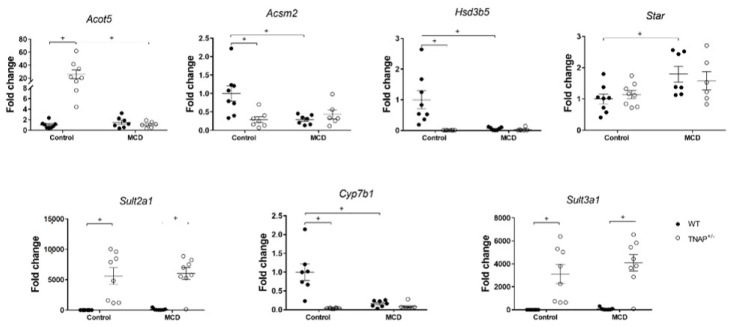
Hepatic expression of genes related to fatty and biliary acid metabolism in wild type (WT) and TNAP heterozygous mice (TNAP^+/−^) fed a control diet (10% *w/w* fat) normalized expression of qRT-PCR data are shown. ^+^ *p* < 0.05.

Next, the expression of key genes involved in hepatic glucidic and fatty acid metabolism was studied using RT-qPCR. As in the mice fed the MCD diet, *Pck1 and Tff3* expression were significantly and profoundly decreased (Figure 4). We next looked at the expression of acyl-Coenzyme A synthetases and acyl-CoA thioesterases, which regulate fatty acid metabolism, namely *Acsm2*, encoding mitochondrial acyl-coenzyme A synthetase, and *Acot5*, encoding an acyl-coenzyme A thioesterase (Figure 6). The expression of *Acsm2* was substantially decreased in the liver of mice fed the MCD diet, but also in haplodeficient mice fed the control diet, and to a similar extent. In turn, *Acot5* was strongly upregulated solely in TNAP^+/−^ mice on the control diet (26-fold, Figure 6). We additionally measured the hepatic expression of hydroxy-delta-5-steroid dehydrogenase (*Hds3b5*), which is involved in steroid metabolism and has been found to be downregulated in steatosis. *Hds3b5* was virtually suppressed in TNAP^+/−^ fed the control diet and the MCD diet (Figure 6).

TNAP has been related to bile acid (BA) homeostasis [15]. Since steatosis has been associated with induction of the acidic BA synthetic pathway, we studied the expression of STAR (*Star*), 25-hydroxycholesterol 7-alpha-hydroxylase (*Cyp7b1*) and sulfotransferases 2a1 and 3a1 (*Sulf2a1* and *Sulf3a1*). STAR (Steroidogenic Acute Regulatory Protein) regulates the first, rate-limiting step in the acidic BA synthetic pathway, generating oxysterols, while CYP7B1 is involved in the next step, which ultimately leads for the most part to the generation of deoxycholic acid [16]. The sulfotransferases in turn are involved in BA conjugation. *Star* was dramatically upregulated in all four groups receiving either the control diet or the MCD diet, more so in the latter (Figure 6 and Figure S2A). We observed a practically total suppression of *Cyp7b1* expression in TNAP^+/−^ vs. WT mice fed the control diet, comparable to that measured in WT and haplodeficient mice fed the MCD diet (Figure 6). Interestingly, our data also show augmented sulfation of BA, based on marked upregulation of *Sulf2a1* and *Sulf3a1*, in haplodeficient mice. Notably, this profile was found to be identical in TNAP^+/−^ mice fed the MCD diet (Figure 6).

No differences in the expression of genes involved in the synthesis of fatty acyl-CoA or bile acid metabolism were observed in mice fed the control vs. the chow diet, indicating that the alterations are due to a genotype:diet interaction (Figure S2B).

### 2.4. Alpl Haplodeficient Mice Exhibit a Reduced Activation of the AMPK but Not the AKT or the PPARα Pathway

**Figure 7:**
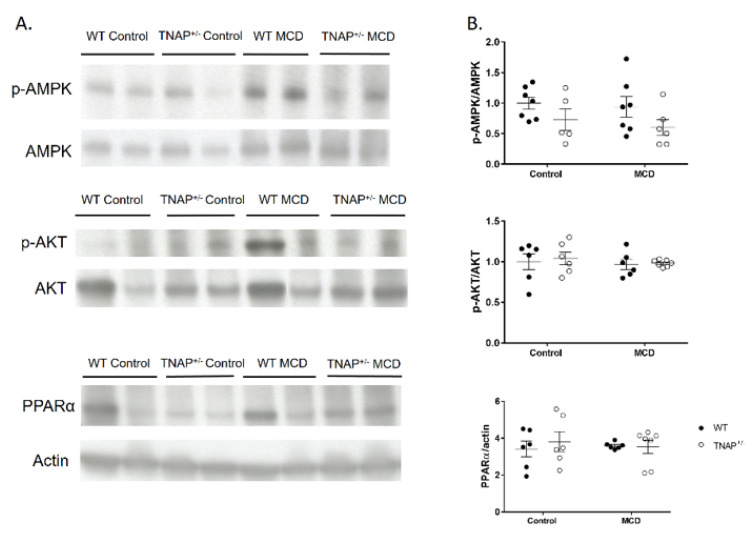
Hepatic ratio of p-AMPK to AMPK, p-AKT to AKT and PPARα to actin in wild type (WT) and TNAP heterozygous mice (TNAP^+/−^) fed a control diet (10% *w/w* fat, control) or the same diet deficient in choline and methionine (MCD). Western blot was used to analyze these parameters. (**A**) Western blot images. (**B**) Quantification.

Because AMPK and AKT phosphorylation and the levels of PPARα have been related to hepatic steatosis, Western blot analysis was carried out with liver samples of WT and TNAP haplodeficient mice fed the MCD or the control diet (Figure 7). A decreased phosphorylated to total AMPK immunoreactivity ratio was observed globally in the liver of haplodeficient mice (ANOVA *p* < 0.05 TNAP^+/−^ vs. WT mice irrespective of diet). However, no statistical differences were noted in AKT phosphorylation or PPARα levels.

## 3. Discussion

The development of NAFLD has been connected to an increase in intestinal permeability that results in endotoxemia, inflammation and a higher fat accumulation in the liver. It has been shown that the administration of a form of alkaline phosphatase, IAP, to mice fed a high fat diet prevents the development of metabolic syndrome and the associated liver damage and steatosis [8]. These effects have been related to its inhibitory effects on fat absorption and endotoxemia [9]. Here, we studied the effect of systemic TNAP haplodeficiency in the development of NAFLD using the MCD model. The latter consists of the administration of a diet devoid of both methionine and choline, resulting in reduced hepatic mitochondrial β-oxidation and compromised very low-density lipoprotein (VLDL) synthesis [11]. Liver expression of TNAP in haplodeficient mice fed a chow diet has been previously shown to be approximately 50% of WT animals at the mRNA level, while AP activity is not substantially diminished, at least using p-nitrophenylphosphate as substrate [7]. In turn, sensitivity to levamisole is altered, consistent with a shift in the glycosylation pattern and therefore of isoform, presumably secondary to changes in biosynthetic rate [17,18].

As previously described, mice fed the MCD diet lost weight progressively, an effect related to increased fatty acid mobilization in extrahepatic tissues. In fact, our experiment had to be discontinued after 17 days for ethical reasons, owing to excessive weight loss. As expected, hypoglycemia was also observed, without glucose intolerance [19]. The MCD diet induced significant fat accumulation in the liver, with no inflammation either locally or at the systemic level. In addition, no intestinal inflammation or alterations in intestinal permeability markers were observed. These data may be due to insufficient follow-up time, as TNF mRNA was already augmented in the liver of MCD-diet-fed mice, albeit nonsignificantly. In fact, our data are consistent with the observation that the MCD diet develops intestinal permeability changes after an initial phase of liver injury and TNF induction [20].

The MCD diet had very similar effects in WT and TNAP^+/−^ mice. In turn, while WT mice fed the control diet exhibited a normal phenotype, TNAP^+/−^ mice unexpectedly developed steatosis, which was actually comparable to that induced by the MCD diet (in either WT or haplodeficient mice). Since no liver anomalies had been previously noted in TNAP^+/−^ mice, liver fat content of mice fed a chow diet (with a regular 4% fat content) was measured. The results obtained confirmed that haplodeficient mice do not develop steatohepatitis in these conditions, indicating that it results from the interaction of TNAP haplodeficiency with the control diet.

Despite a similar degree of hepatic steatosis, several features were different between the TNAP^+/−^ mice fed the control diet of the MCD diet. Thus, the livers of the former presented numerous very small lipid vesicles (i.e., microsteatosis), while the livers of MCD fed mice had predominantly large lipid droplets (i.e., macrosteatosis). In addition, ALT levels were increased only in MCD-fed mice, while hepatic *Il1b* gene expression was upregulated exclusively in TNAP^+/−^ mice fed the control diet (with *Tnf* closely following, albeit nonsignificantly, as mentioned). Similarly, *Des* (and *Tgfb1* nonsignificantly) was increased in TNAP^+/−^ control mice, pointing to augmented fibrosis. To gain further insight on the features of liver steatosis induced by the control diet in TNAP-deficient mice, we characterized basic genes involved in the glucidic and lipid metabolism. Data show an expression profile akin to that in the MCD diet groups. Thus *Pck1*, encoding cytoplasmatic PEPCK, the key enzyme in gluconeogenesis, was dramatically downregulated, consistent with inhibition of the citric acid cycle and fatty acid accumulation [21]. In contrast to other studies indicating a reduced expression of genes involved in fatty acid synthesis and desaturation [22], we found no changes in *Fadsn*, *Acaca, Scd1, Fads1* and *Fads2* in any of the groups. In order to participate in metabolic processes (oxidation and synthesis of complex lipids), fatty acids are activated by reaction with CoA to form fatty acyl CoA. This reaction is catalyzed by different fatty acid acyl-CoA synthetases, while acyl-CoA thioesterases catalyze the deactivation of fatty acid acyl-CoA. The expression of *Acsm2*, encoding mitochondrial acyl-coenzyme A synthetase for medium-chain fatty acids 2, which has been related to insulin resistance [23], was inhibited in the MCD diet groups as well as in TNAP^+/−^ control mice, while that of *Acot5*, encoding an acyl-coenzyme A thioesterase, was enhanced only in TNAP^+/−^ control mice. These data are consistent with reduced fatty acid Acyl-CoA synthesis by MCD diet and TNAP haplodeficiency with a control diet, resulting in their lipid accumulation, reinforced by enhanced deactivation of fatty acid Acyl-CoA in TNAP^+/−^/control mice.

Moderate elevations of serum levels of TNAP and total BA are common in NAFLD/NASH patients [24,25]. Steatosis is associated with induction of the acidic BA biosynthetic pathway, which is normally subdued (<10%) [16]. In addition, TNAP is induced in cholestasis, although the role of TNAP in BA homeostasis is poorly characterized [15]. Strong induction of *Star*, which is considered the rate-limiting step in the acidic BA pathway, was noted in all four groups receiving control/MCD diets. However, steatosis did not develop in WT mice on a control diet. The acidic pathway has been shown to lead to a smaller BA pool with increased hydrophilicity and lower cholesterol dietary absorption and improved lipid homeostasis. However, the downregulation of *Cyp7b1* in this pathway, which is typically found in steatosis [26], results in the accumulation of oxysterol intermediaries, which have important inflammatory and regulatory effects. Of note, *Cyp7b1* expression was strongly and inversely correlated with steatosis in the control/MCD groups. Interestingly, it was induced in WT mice on a control diet, suggesting a protective role in this context. *Cyp7b1* expression is negatively regulated by insulin resistance, which was not present in our study, and also by BA stimulation of FXR [16]. The expression profile was very similar in the case of *Hsd3b5*, which is involved in steroid metabolism and is also downregulated in steatosis. Thus BA accumulation may be a common mechanism in TNAP^+/−^/control diet and MCD mice.

Interestingly, the expression of the sulfotransferases *Sult2a1* and *Sult3a1* was induced in TNAP haplodeficient mice fed a control diet, independently of choline and methionine content, but not with a standard diet. Sulfation is a conjugation reaction catalyzed by sulfotransferases whereby a sulfonate (SO_3_^−^) group is transferred from the universal sulfonate donor 3′-phosphoadenosine 5′-phosphosulfate (PAPS) to a substrate. BA sulfation is favored in cholestasis [27], and it increases renal and intestinal elimination of BA. In mice, sulfation involves the C7 position, which is less prone to hydrolysis [28]. These data are consistent with enhancement of BA elimination by TNAP haplodeficiency, a TNAP^+/−^/control-diet-specific effect that cannot be ascribed to modulation of the BA acidic pathway, choline depletion or steatosis.

**Figure 8:**
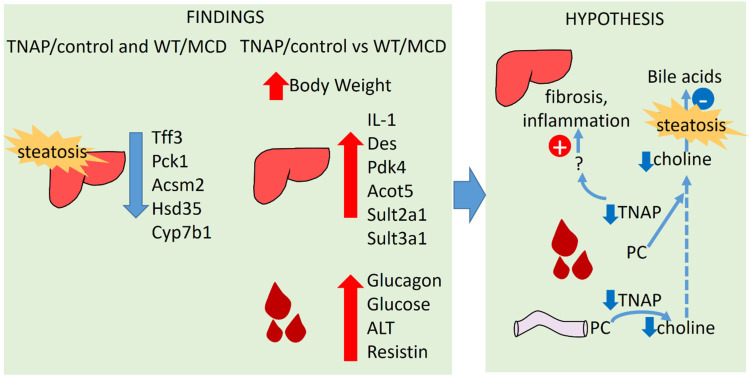
Hypothesis based on the findings of the study.

Our data therefore show that steatosis results from both methionine and choline deficiency and from TNAP haplodeficiency when exposed to a control diet (Figure 8). No changes were observed in mice fed the chow diet, indicating an interaction between TNAP and the control diet. The phenotype is more severe in the former, probably because the MCD diet produces peripheral fat mobilization, but there is clear overlap (i.e., TNAP^+/−^ mice are similar to WT mice). This suggests a possible common pathogenic mechanism. In this regard, TNAP may play a role in choline absorption by dephosphorylation of phosphocholine. It is possible therefore that choline deficiency may be generated in TNAP haplodeficient mice. Another possibility is the disruption of transcellular lipid transport as described for IAP KO mice in the intestine [9]. The fact that such a striking phenotype results from the lack of a single allele is remarkable, particularly considering that alkaline phosphatase activity is not significantly reduced in the liver of TNAP^+/−^ mice, as observed also in a previous study [7]. As these measurements have been carried out using a standard technique with p-nitrophenylphoshate as substrate, it is possible that activity varies with other substrates, due to changes in glycosylation, as stated above. It is interesting that Alpl mRNA expression was upregulated in TNAP^+/−^/control diet mice (despite the lack of one allele), whereas alkaline phosphatase activity was increased in both MCD groups, with different sensitivities to levamisole. Taken together, these data point to modulation of TNAP in the three steatotic groups, perhaps secondary to BA accumulation. These mechanisms will need future confirmation.

In conclusion, in this study, we have shown that the deficiency in TNAP associated with a control diet containing 10% *w/w* of fat induces a steatogenic response similar to that observed in MCD-fed WT animals, with increased inflammatory and fibrotic features and specific alterations in BA excretion.

## 4. Materials and Methods

### 4.1. Animals and Experimental Design

C57BL/6 heterozygous male and female mice for Alpl (B6.129S7-Akp2tm1Sor/J, referred to as TNAP^+/−^ mice) and wild-type (WT) littermates as controls were used in the study. Seven to nine mice per group were studied. Mice were maintained at the Unit of Animal Research (Biomedical Research Center, University of Granada, Granada, Spain) in air-conditioned animal quarters with a 12 h light/dark cycle and specific pathogen-free conditions. Animals were housed in Makrolon^®^ cages (4–5 mice per cage, males and females kept separately) and were given free access to autoclaved tap water and food (Harlan-Teklad 2014, Harlan Ibérica, Barcelona, Spain) until the first day of the study. Animal status was monitored daily for signs of pain or suffering. Predefined criteria for euthanasia included major weight loss, lack of movement and huddling behavior (none of the latter were detected). Mice were anesthetized with an intraperitoneal injection of a ketamine/xylazine mixture prior to cardiac exsanguination. All protocols were approved by the Animal Welfare Committee of the University of Granada (registry number: CEEA 01/03/2017–029) and carried out according to the Guide for the Care and Use of Laboratory Animals. This study complies with the requirements on reporting research detailed in the ARRIVE guidelines.

WT and TNAP^+/−^ mice were distributed randomly to receive the MCD or the control diet (see below). Thus a 2 × 2 design was followed. Treatment was not blinded. Mice weights were recorded daily. Animals were fed ad libitum for 17 days, when weight loss reached the ethical limit in the MCD groups.

### 4.2. Diets

The methionine- and choline-deficient diet (Ref. TD.90262) and the control diet (Ref. TD.94149) were purchased from Envigo (Indianapolis, IN, USA). Composition is given in Table 1 and Table 2. In some measurements, samples obtained from previous experiments with chow-fed animals were used as a reference group. After a 12 h fast, mice were euthanized, and plasma samples and liver tissue fragments were collected and stored at −80 °C.

### 4.3. Histological Assessment

Liver tissue fragments were fixed in 4% paraformaldehyde and embedded in paraffin. For histological evaluation, slides were stained with hematoxylin and eosin (H&E). Images were taken and digitalized using a Leica DMI3000B microscope (Leica, Wetzlar, Germany) equipped with a Leica DFC420 camera. Sections were scored blindly for fat-drops size and frequency.

### 4.4. Plasmatic Parameters

Fasting plasma samples were assayed for alanine aminotransferase activity (Sigma-Aldrich, San Luis, MO, USA) and glucose concentration. The glucose oxidase method was used. Plasmatic parameters were measured using Metabolic Hormone Expanded Panel multiplex (ref. MMHE-44K, Merk Millipore, Burlington, MA, USA) according to manufacturer’s instructions. HOMA-IR was calculated using the formula:

$$HOMA-IR=\frac{glucose\;\left(\frac{mg}{dL}\right)\times insulin}{405}$$

### 4.5. Alkaline Phosphatase Activity

Colonic and hepatic tissues were mechanically homogenized in HTAB (Hexadecyltrimethylammonium Bromide) buffer using a Bullet Blender (Next Advance, Averill Park, NW, USA). Alkaline phosphatase activity was measured spectrophotometrically, using 5.5 mM disodium nitrophenyl phosphate as substrate. Sensitivity to the specific inhibitor levamisole was tested and expressed as inhibition percentage.

### 4.6. Hepatic Fat Content

Liver samples were homogenized in distilled water using an electric homogenizer (Heidolph, Schwabach, Germany). Chloroform:methanol solution was added to the homogenates to a 1:1:1 mix that was vortexed and centrifuged at 1100 g/20 min/4 °C. The organic phase was collected and dried under a stream of nitrogen. Dry extract was weighed, and results were reported as mg fat per gram of liver.

### 4.7. RNA Isolation and Quantitative Reverse-Transcription Polymerase Chain Reaction (RT-qPCR) Analysis

RNA was isolated using RNeasy MiniKit (Qiagen, CA, USA) following the manufacturer’s instructions. RNA quantity and integrity were assessed spectrophotometrically with Nanodrop (Thermo Fisher Scientific, Waltham, MA, USA). Total RNA was subjected to reverse transcription (BioRad, Hercules, CA, USA), and resulting cDNA was subjected to quantitative PCR (Promega, Madison, WI, USA). Primers used to amplify *Srebf1*, *Fasn* and *Acaca* were predesign primers from MilliporeSigma (Madrid, Spain). Other primers used, and also purchased from MilliporeSigma, are shown in Table S1.

### 4.8. Western Blot

Liver tissues were homogenized in lysis buffer (0.1% *w*/*v* SDS, 0.1% *w*/*v* sodium deoxycholate, 1% *v/v* Triton X-100 in PBS) with a protease inhibitor cocktail 1:200 (*v/v*) (P9599; MilliporeSigma, Madrid, Spain) and a phosphatase inhibitor cocktail 1:100 (*v/v*) (SC-45045; Santa Cruz Biotechnology, Dallas, TX, USA) using a Bullet Blender (Next Advance, Averill Park, NW, USA). Homogenates were sonicated and centrifuged 12,000 g/10 min/4 °C. After protein concentration was determined by the bicinchoninic acid assay [29], samples were heated at 95 °C for 5 min in Laemmli sample buffer (BioRad, Hercules, CA, USA). Proteins were separated by SDS-PAGE and transferred onto nitrocellulose membranes. Samples were immunoblotted using the primary antibodies rabbit anti-AKT (ref. 9272s, Cell Signaling Technology, MA, USA), Ser473 rabbit anti-pAKT antibody (ref. 4060s, Cell Signaling Technology, MA, USA), rabbit anti-AMPKα antibody (ref. 2532L, Cell Signaling Technology, MA, USA), Thr172 rabbit anti-pAMPKα antibody (ref. 2535s, Cell Signaling Technology, MA, USA), rabbit anti-PPARα antibody (ref. ab3484, Abcam, Cambridge, UK) and mouse anti-actin (A5441, Sigma-Aldrich, San Luis, MO, USA) at 1:1000 dilution. After incubation with a secondary antibody, bands were detected by ECL (PerkinElmer, Waltham, MA, USA) and quantified using ImageJ (National Institutes of Health, USA).

### 4.9. Statistical Analysis

Samples were run at least in triplicate, and results were expressed as mean ± standard error of the mean (SEM). Differences among means were tested by two-way ANOVA and a posteriori Tukey’s multiple comparisons test or Student’s *t*-test for pairwise comparisons. Analyses were carried out with GraphPad Prism 7 software (GraphPad Software, San Diego CA, USA). Significance was accepted at *p* ≤ 0.05.

## Figures and Tables

**Table 1:** Composition of diets.

	Control	MCD Diet	Chow Diet
Sucrose (g/kg)	455	455	445
Corn starch (g/kg)	200	200	200
Corn Oil (g/kg)	100	100	40
l-Methionine (g/kg)	8.2	-	8.2
Choline (g/kg)	1.4	-	1.4

**Table 2:** Elemental composition of MCD and control diets.

g/Kg	Control Diet	MCD Diet
Sucrose	443.597	455.294
Corn starch	198.783	200.0
Corn Oil	100.0	100.0
Cellulose	30.0	30.0
Mineral Mix, AIN-76 (170915)	35.0	35.0
Calcium Phosphate, dibasic	3.0	3.0
L-Alanine	3.5	3.5
L-Arginine HCl	12.1	12.1
L-Asparagine	6.0	6.0
L-Aspartic Acid	3.5	3.5
L-Cysteine	3.5	3.5
L-Glutamic Acid	40.0	40.0
Glycine	23.3	23.3
L-Histidine HCl, monohydrate	4.5	4.5
L-Isoleucine	8.2	8.2
L-Leucine	11.1	11.1
L-Lysine HCl	18.0	18.0
L-Methionine	8.2	-
L-Phenylalanine	7.5	7.5
L-Proline	3.5	3.5
L-Serine	3.5	3.5
L-Threonine	8.2	8.2
L-Tryptophan	1.8	1.8
L-Tyrosine	5.0	5.0
L-Valine	8.2	8.2
Vitamin Mix, Teklad (400600) + Choline dihydrogen citrate	10.0 (1.4)	-
Vitamin Mix, w/o choline, A, D, E (83171)	-	5.0
Vitamin E, DL-α tocopherol acetate (500 IU/g)	-	0.242
Vitamin A Palmitate (500,000 IU/g)	-	0.0396
Vitamin D3, cholecalciferol (500,000 IU/g)	-	0.0044
Ethoxyquin, antioxidant	0.02	0.02

## Supplementary Materials

Supplementary materials can be found at https://www.mdpi.com/1422-0067/22/1/51/s1.

## Abbreviations

ALT	alanine aminotransferase
BA	bile acids
GIP	gastric inhibitory peptide
GLP-1	glucagon-like peptide 1
HOMA-IR	homeostasis model assessment–insulin resistance
IAP	intestinal alkaline phosphatase
MCD	Methionine- and choline- deficient diet
NAFLD	nonalcoholic fatty liver disease
NASH	nonalcoholic steatohepatitis
PC	Phosphorylcholine
PAP	pancreatic peptide
PEPCK	phosphoenolpyruvate carboxykinase
PYY	peptide YY
TNAP	tissue nonspecific alkaline phosphatase
VLDL	very low-density lipoproteins
