# Supplementary material for: Deficiency in Tissue Non-Specific Alkaline Phosphatase Leads to Steatohepatitis in Mice Fed a High Fat Diet Similar to That Produced by a Methionine and Choline Deficient Diet
Source: Int J Mol Sci. 2020 Dec 23;22(1):51. doi: 10.3390/ijms22010051 (PMC7793076; doi:10.3390/ijms22010051)
Supplement: Supplementary file 1 [file ijms-22-00051-s001.pdf]

Table S1. Primers used in PCR analysis.

Gene	Sense primer	Antisense primer
18s	ACACGGACAGGATTGACAGATTG	GCCAGAGTCTCGTTCGTTATCG
Acot5	ATCTGGAGTACTTTGAGGAAG	TTGAAATAACGTTGACCGTG
Acsm2	ACAATGAAAGTCAAACCAGG	GATTGTCCACATATCCAGAG
Alpl	ATTCCCACTATGTCTGGAAC	CTCAAAGAGACCTAAGAGGTAG
Cd14	GAATCTACCGACCATGGAG	AAGTTGCAGGAACAACTTTC
Cldn4	GACTGTGCAAAGTTACTAGC	ACCAGCAATTTGGATGTAAG
Des	CAGGATCAACCTTCCTATCC	CTGTCTTTTGGTATGGACTTC
Cyp7b1	ATGCTCCAAAGGAATTTAGG	TCCAAAAGGCATAACGTAAG
G6pc	TTCAAGTGGATTCTGTTTGG	AGATAGCAAGAGTAGAAGTGAC
Fads1	TTCTATTTTGTGGTTCAGCG	ATGGGGATATGGTTCATCTG
Fads2	TCATCATGACAATGATCAGC	CAGGAACCTGATAAAGTTGAG
Glut2	TTGTGCTGCTGGATAAATTC	AAATTCAGCAACCATGAACC
Gpx1	GGAGAATGGCAAGAATGAAG	TTCGCACTTCTCAAACAATG
Hprt	AGGGATTTGAATCACGTTTG	TTTACTGGCAACATCAACAG
Hsd3b5	CATGAAGAGGAATTGTCCAAG	GCTGAGTACCTTTCAGATTG
Il1b	GGATGATGATGATAACCTGC	CATGGAGAATATCACTTGTTGG
Il6	AAGAAATGATGGATGCTACC	GAGTTTCTGTATCTCTCTGAAG
Lbp	CACTCCCCAGATATACAAAAAG	AGGCAAATACATTAGTGACC
Panx1	AGTTCAAAGATTTGGACCTG	GACACACACTACATCAATACC
Pck1	AATATGACAACTGTTGGCTG	AATGCTTTCTCAAAGTCCTC
Pdk4	ACAATCAAGATTTCTGACCG	TCTCCTTGAAAATACTTGGC
Scd1	GTGGGGTAATTATTTGTGACC	TTTTTCCCAGACAGTACCAAC
S100a8	GATGGTGATAAAAGTGGGTG	CTGTAGACATATCCAGGGAC
Spp1	GGATGAATCTGACGAATCTC	GCATCAGGATACTGTTCATC
Star	GCGGAATATGAAAGGATTAAGG	GTCACTATAGAGTGTTGCTTC
Sult2a1	GTCTTTCTTCAGTTCCAAGG	CCCAGAAAAAGTAACCAGAC
Sult3a1	TTCTAGATGGAGATGTGGTAG	CTTCACTCAGTTCTTTCTCC
Tff3	CCTGGTTGCTGGGTCCTCTG	GCCACGGTTGTTACACTGCTC
Tgfb1	CCCTATTTAAGAACACCC	AAGGTGTCTCAGTATACCAC
Tnf	CGTGGAACTGGCAGAAGAGG	CAGGAATGAGAAGAGGCTGAGAC
Tjp1	GGGGCCTACACTGATCAAGA	TGGAGATGAGGCTTCTGCTT

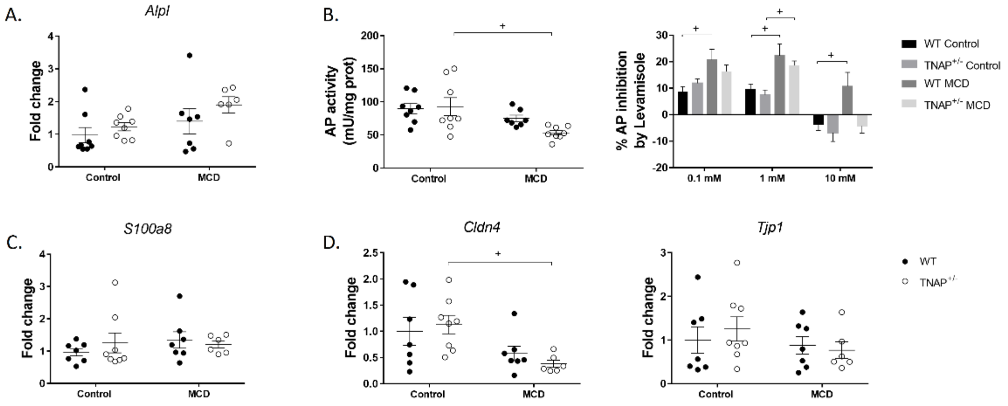

Figure S1. Intestinal inflammatory and barrier markers in wild type (WT) and TNAP heterozygous mice (TNAP$^{+/-}$) fed a control diet (10% w/w fat, control) or the same diet deficient in choline and methionine (MCD). A. *Alpl* expression (RT-qPCR). B. AP activity and inhibition by levamisole. C. *S100a8* expression (RT-qPCR). D. Claudin 4 and ZO1 expression by RT-qPCR.

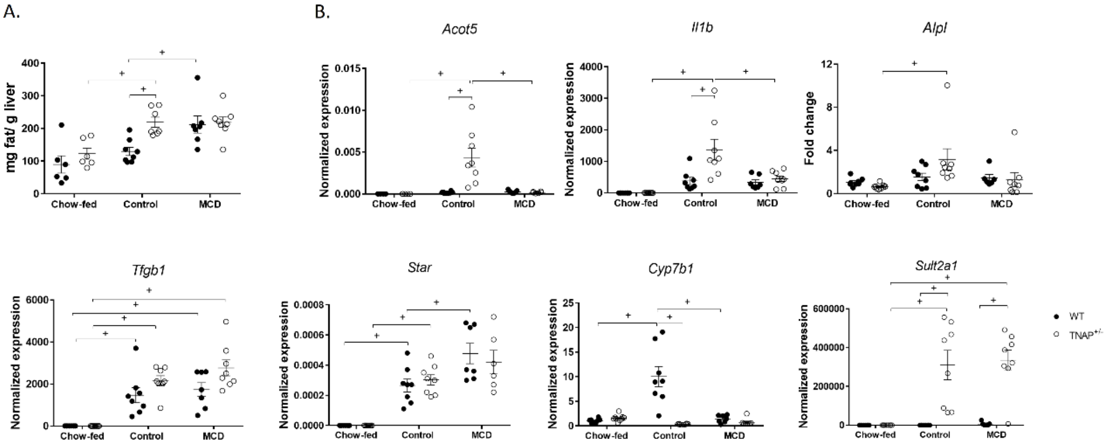

Figure S2. Comparation of hepatic parameters between the study groups and reference wild type (WT) and TNAP heterozygous mice (TNAP$^{+/-}$) fed regular chow diet. A. Liver fat content. B. Expression of various genes by RT-qPCR. $^{+}p<0.05$.
